# Determinants of sugar‐sweetened beverage consumption in young children: a systematic review

**DOI:** 10.1111/obr.12310

**Published:** 2015-08-07

**Authors:** V. Mazarello Paes, K. Hesketh, C. O'Malley, H. Moore, C. Summerbell, S. Griffin, E. M. F. van Sluijs, K. K. Ong, R. Lakshman

**Affiliations:** ^1^ Institute of Public HealthUniversity of CambridgeCambridgeUK; ^2^ Institute of Child HealthUniversity College LondonLondonUK; ^3^ MRC Epidemiology Unit and UKCRC Centre for Diet and Activity Research (CEDAR)University of CambridgeCambridgeUK; ^4^ School of Medicine, Pharmacy and HealthDurham University Queens CampusStockton‐On‐TeesUK

**Keywords:** Correlates, determinants, sugar‐sweetened beverage, systematic review, young children

## Abstract

Sugar‐sweetened beverage (SSB) consumption is associated with adverse health outcomes. Improved understanding of the determinants will inform effective interventions to reduce SSB consumption. A total of 46,876 papers were identified through searching eight electronic databases. Evidence from intervention (*n* = 13), prospective (*n* = 6) and cross‐sectional (*n* = 25) studies on correlates/determinants of SSB consumption was quality assessed and synthesized. Twelve correlates/determinants were associated with higher SSB consumption (child's preference for SSBs, TV viewing/screen time and snack consumption; parents' lower socioeconomic status, lower age, SSB consumption, formula milk feeding, early introduction of solids, using food as rewards, parental‐perceived barriers, attending out‐of‐home care and living near a fast food/convenience store). Five correlates/determinants were associated with lower SSB consumption (parental positive modelling, parents' married/co‐habiting, school nutrition policy, staff skills and supermarket nearby). There was equivocal evidence for child's age and knowledge, parental knowledge, skills, rules/restrictions and home SSB availability. Eight intervention studies targeted multi‐level (child, parents, childcare/preschool setting) determinants; four were effective. Four intervention studies targeted parental determinants; two were effective. One (effective) intervention targeted the preschool environment. There is consistent evidence to support potentially modifiable correlates/determinants of SSB consumption in young children acting at parental (modelling), child (TV viewing) and environmental (school policy) levels.

**Abbreviations**
BMI: body mass index
EPPI: evidence for policy and practice information
PROSPERO: International Prospective Register for Systematic Reviews
SSB: sugar‐sweetened beverage
XS: cross‐sectional

## Introduction

Forty‐three million children aged 0–5 years are obese or overweight worldwide, and the prevalence of obesity in children is estimated to rise from 4.2% in 1990 to 9.1% in 2020 1. Childhood obesity has important consequences for health and well‐being during childhood and also in later adult life 2. According to the National Child Measurement Programme for England, over a fifth of children (22.2%) aged 4–5 years were overweight or obese on school entry. In the final year of primary school, one in three children (33.3%) aged 10–11 years was obese or overweight 3. However, levels of obesity have begun to plateau in Australia, United States and many European countries including United Kingdom 4, 5, 6, 7, 8, 9. A recent study in the United States reported that a child's weight status is set by age 5 and tracks throughout childhood, as nearly half of children who became obese by the eighth grade were already overweight when they started school 10.

Several cross‐sectional (XS) 11, 12 and prospective studies 13, 14 have described the association between sugar‐sweetened beverage (SSB) consumption and obesity in young children. Moreover, recent systematic reviews on experimental evidence show that reducing SSB consumption in young children is successful in reducing obesity 15, 16. Studies additionally show that consumption of SSBs in young children is a risk factor for overall poor diet quality 17 and oral health 18, 19. In addition to weight gain and poor oral health, higher consumption of SSBs is associated with development of metabolic syndrome and cardio‐metabolic risk factors such as type 2 diabetes later in life 20, 21, 22.

Therefore, the rising prevalence of childhood obesity poses a major public health challenge in both developed and developing countries due to the increasing burden of chronic non‐communicable diseases 23. Amid controversy with regard to the role of SSB consumption in obesity development/weight gain, there is growing evidence to suggest that decreasing SSB consumption will reduce the prevalence of obesity and obesity‐related chronic diseases. Meanwhile, despite resistance from the beverage industry, several public policies and regulatory strategies to reduce consumption of SSBs are already in place or being developed worldwide 15, 24, 25, 26.

Evidence suggests that unhealthy dietary habits such as SSB consumption are formed during early childhood and stress the need to understand the correlates/determinants influencing these behaviours in children to inform intervention development 27, 28. The socio‐ecological model of health behaviour suggests that an individual's behaviour is influenced by a multitude of correlates/determinants operating at different levels. This systematic review synthesizes quantitative evidence from intervention and observational (prospective cohort and XS) studies on the determinants and correlates of SSB consumption in young children (0–6 years) using the socio‐ecological model.

## Methods

In the absence of a standard definition for SSBs, for the purpose of this review, we defined SSBs as beverages that are high in added sugar and add calories to diet 29, 30. The definition includes sweetened milk (flavoured milk or milk alternatives), fruit drinks (sweetened fruit juice), soft drinks (bottles or cans of non‐alcoholic, flavoured, carbonated or non‐carbonated beverages), tea and coffee drinks (sweetened), energy drinks, sports drinks and any other beverages to which sugar (high‐fructose corn syrup, sucrose or table sugar) has been added.

This review is part of a series of systematic reviews of quantitative and qualitative evidence on determinants of obesogenic behaviours in young children (International Prospective Register for Systematic Reviews [PROSPERO] Registration number: CRD42012002881). The overall study design, search and quality assessment strategies are previously described in the published protocol 31. Methods follow those described for the rigorous conduct and reporting of systematic reviews for policy and practice published by the Evidence for Policy and Practice Information (EPPI) Centre 32.

### Search strategy, inclusion/exclusion and quality assessment criteria

Further to an iterative scoping stage, with input from experts, a combined search strategy with terms related to population (young children aged 0–6 years), exposure and outcome (fruit and vegetable consumption, SSB and other obesogenic diet consumption, physical activity and sedentary behaviours) were used to identify papers (details in protocol paper 31). Eight electronic databases were searched from inception to June 2014. No language restrictions were applied, but clinical populations were excluded. All identified articles were imported into an Endnote database and after de‐duplication a total yield of 46,876 articles was achieved. Specifically for SSB consumption behaviours, the inclusion/exclusion criteria have been described in Table 1. The quality assessment criteria were based on methods described by the EPPI Centre 32 and presented in Table 2. For intervention studies, eight items were scored focusing on internal validity (e.g. randomization procedure, objective measure of outcome, retention). For observational studies, six items were scored focusing on both internal and external validity. Studies were classified as high, intermediate or low quality based on the number of quality criteria met (for intervention studies: low: ≤2; intermediate: 3–5; high: ≥6; for observational studies: low: ≤2; intermediate: 3–4; high: ≥5).

**Table 1:** Inclusion and exclusion criteria for determinants of SSB consumption review in young children

Inclusion criteria	Exclusion criteria
Interventional studies (RCTs and non‐RCTs) targeting SSB consumption Non‐intervention/observational, i.e. cohort and cross‐sectional (XS) studies that quantified the association between correlate/determinant AND SSB consumption in obese or non‐obese children Studies that measured SSB consumption (diet diaries, food records, 24‐h recalls, questionnaires) Children aged less than 7 years at baseline	Non‐human studies Laboratory‐based (such as vitamin and preloading studies) Studies on health outcomes for these behaviours (i.e. studies describing the association between dietary habits and obesity or cardiovascular risk factors) Studies not reporting consumption data Quantitative studies that measured SSB behaviours but did not describe an association Studies in clinical populations (e.g. malnutrition, disability, allergy, dental caries, asthma, cerebral palsy, cystic fibrosis, autism) Studies on breast/bottle feeding and weaning in infants with no association with SSB consumption described

RCT, randomized controlled trial; SSB, sugar‐sweetened beverage.

**Table 2:** Quality assessment criteria by study design for review of determinants of SSB consumption in young children

For intervention studies	For observational (prospective cohort and cross‐sectional) studies
Total quality assessment score (maximum of eight) was derived for fulfilment of the following criteria Randomization Effect of intervention reported for all outcomes Pre‐intervention data on all outcomes Post‐intervention data on all outcomes Allocation concealment Blinding Objective measurement of outcome Retention >70% Studies with small sample size (*n* < 50) and no control group were considered to provide lower quality evidence and not scored.	Total quality assessment score (maximum of six) was derived for fulfilment of the following criteria More than 50 participants analysed Studies representing general population Prospective study design Adjusted/multivariate analysis Objective measure of outcome Objective measure of exposure

SSB, sugar‐sweetened beverage.

### Study selection

Duplicate review of at least a subsample of papers was carried out at each stage of the review process: title and abstract screening (CO, KH, VMP, CS, EMFvS and RL), data extraction (VMP, EMFvS, KKO and RL) and quality assessment (VMP, EMFvS, KKO and RL) to ensure high level of agreement and minimize any reviewer‐related biases.

Full texts of articles appearing to meet the inclusion criteria were retrieved for further review and their status recorded in a pre‐piloted IN/OUT spreadsheet, along with specific study details and reasons for exclusion (for excluded studies). Foreign language papers were translated by native speakers. Articles were re‐examined (CS, EMFvS or RL) if there was uncertainty about inclusion criteria and disagreements were resolved at team meetings.

**Figure 1:**
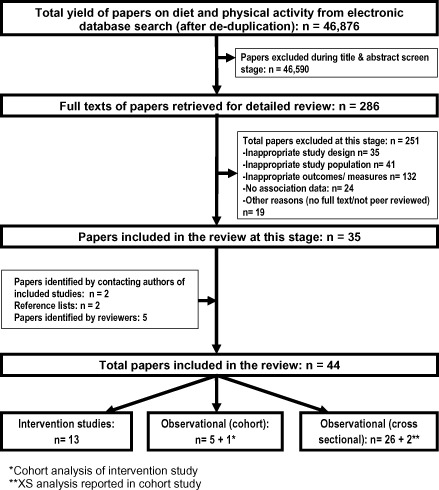
Overview of search results for evidence on determinants of sugar‐sweetened beverage consumption in young children.

A total of 286 full‐text papers related to SSB and obesogenic dietary behaviours were identified for further review, of which 35 papers met the inclusion criteria. Nine additional papers were identified, two 33, 34 through correspondence with first authors of included studies and two 35, 36 through hand searching/citation tracking of reference lists of included studies (additionally, five were identified by reviewers). A summary flow chart of the literature identification strategy is presented in Fig. 1.

### Data extraction

Extracted data (Supplementary Table S1) from all included papers were entered into a previously piloted data extraction spreadsheet and analysed by one reviewer (VMP). Additional to the included articles, cohort data from control group participants were extracted for one intervention study 34, and two prospective studies also reported on XS associations 37, 38. These three studies have been presented and accounted for as per study design (Fig. 1). Multiple papers reporting data from the same study were extracted and referenced separately but considered as one study.

‘Intervention effect’ was defined as the reported difference in SSB consumption in intervention vs. control groups at follow‐up (adjusted for baseline consumption where relevant); the intervention was considered to have a positive effect if it reduced SSB consumption compared with control group. From prospective cohort studies, the association between the determinant at baseline and change in SSB consumption between baseline and follow‐up was extracted. From XS studies, the reported association between the correlate and SSB consumption at the same time point was extracted.

For quality assurance, all the intervention (*n* = 13) and cohort (*n* = 6) studies and a sample of over 10% of XS studies (*n* = 25) were double reviewed by a second reviewer (RL) to ensure agreement and consistency in data extraction and reporting. Furthermore, all intervention studies (*n* = 13) were double reviewed and analysed (EMFvS and KKO) to identify target determinants.

### Data synthesis

A narrative synthesis (including tables) was undertaken. Because of the heterogeneity between studies (in study quality/design, setting, participant characteristics, behavioural models, measures of correlates/determinants and behaviours) and analyses (proportions, means, beta‐coefficients and odds ratios), meta‐analysis was not appropriate.

We performed a non‐quantitative synthesis of all reported determinants or correlates of SSB consumption grouped according to the levels of the socio‐ecological model. Conceptually similar exposures were combined. For each potential determinant, findings from individual studies were categorized as ‘−’ significantly lower/decreased SSB consumption, ‘0’ no significant association/effect or ‘+’ significantly higher/increased SSB consumption. Consistency across studies was then summarized using a previously applied algorithm 39, 40 labelled as ‘0’ (no association) if supported by 0–33% of individual studies, ‘?’ (indeterminate/possible) if supported by 34–59% and ‘+’ or ‘−’ if supported by 60–100%. Moreover, where four or more studies reported on a potential determinant, double signs were used to indicate greater confidence in the summary (e.g. ‘00’, ‘??’, ‘++’ and ‘−−’).

## Results

### Study characteristics

#### Intervention studies

Reports from 13 intervention studies, published between 2007 and 2013, were identified (Supplementary Table S2). Four studies were conducted in the Americas, four in Australia, four in Europe (Belgium *n* = 2, Spain *n* = 1, United Kingdom *n* = 1) and one in Asia. Children's age ranges varied from early infancy to 4–6 years old. Duration of interventions varied from 8 weeks to 4 years, and post‐intervention follow‐up was either immediate (*n* = 11), 6 months (*n* = 1) or 4 years (*n* = 1). Seven intervention studies used a behaviour change theory, four of which showed a significant positive effect in favour of the intervention. Of the six interventions that did not use a behaviour change theory, three studies showed a significant positive effect.

Eight intervention studies 34, 41, 42, 43, 44, 45, 46, 47 targeted multi‐level (child, parents, childcare/preschool setting) determinants of SSB consumption, of which four 43, 44, 46, 47 reduced SSB consumption. Four interventions 33, 48, 49, 50, 51 (one intervention 48, 49 reported results at two time points) exclusively targeted parental determinants and two 33, 51 reduced SSB consumption. One study 52 targeted the preschool environment and reduced SSB servings in the child's lunchbox. No interventions exclusively targeted child determinants of SSB consumption. No intervention studies reported on a mediation analysis to suggest that a change in any particular determinant was associated with change in SSB consumption. Effective studies were set outside of Europe (Australia *n* = 3, Asia *n* = 1, United States *n* = 3). A UK study did not show a significant effect post‐intervention (age 1 year), but reported a significant positive effect at age 4 years 48, 49. Seven studies 33, 45, 46, 47, 48, 49, 50, 51 including one very small pilot study 46 recruited non‐representative populations. Five studies were rated as ‘high’ quality 44, 45, 48, 49, 51, 52; three of these reported significant beneficial effects of the intervention 44, 49, 52.

#### Observational – prospective cohort studies

Six prospective cohort studies published between 1999 and 2012 were identified (Supplementary Table S3). Three studies were set in the United States 38, 53, 54 and one each in Australia 37, Belgium 34 and Germany 55. Four were in non‐representative populations: two in populations with limited income 28, 43, one with health conscious participants 55 and one had a small sample of Caucasian infants (*n* = 49) 54. The studies had an average follow‐up period of 2 years and the age range varied from infants up to 6.5 years old. The studies were of intermediate (*n* = 4) or low (*n* = 2) quality and investigated a total of seven determinants: three at the individual level (child) and four at the interpersonal (parent/caregiver) level.

#### Observational – XS studies

We identified 25 XS studies of intermediate (*n* = 19) and low (*n* = 6) quality published between 2002 and 2013 (Supplementary Table S4). Nine studies were conducted in the United States 38, 56, 57, 58, 59, 60, 61, 62, 63, two in Canada 36, 64, 65 and one in both the United States and Mexico 66. Three studies 37, 67, 68 were from Australia and 10 from Europe (two 69, 70 from Belgium, one each from Spain 71, 72, Finland 73, Sweden 74, the Netherlands 75, 76, one 77 in five European countries and three 35, 78, 79, 80, 81 from the United Kingdom). The populations varied across studies (some had infant populations while others were in 2.5‐ to 7‐year‐old children) and eight 35, 38, 59, 60, 61, 62, 66, 79 were in non‐representative populations.

### Summary of correlates/determinants of SSB consumption

Evidence from the intervention and observational (prospective and XS) studies was pooled to identify potential determinants of young children's SSB consumption, according to levels of the socio‐ecological model (Table 3). There was little overlap between determinants targeted in intervention studies (taste exposure, knowledge, attitudes, motivation, perceived barriers, encouragement/support, skills, policy and availability) and correlates/determinants in observational studies. Of the 54 correlates/determinants, only child's age and parents' socioeconomic status and SSB consumption were determinants identified in prospective studies.

**Table 3:** Potential correlates/determinants of young children's (≤6 years) sugar‐sweetened beverage consumption

Correlate/determinant	Association with SSB consumption*			No. of studies	Summary[Link], [Link]
	−	0	+		
Individual (child)					
Sex (ref: girls)		*C5, C12, C19*	*C6*	1/4	00
Age		P3, P5, P6, *C19, C23*	P4, *C2, C5, C6, C22*	5/10	??
Knowledge	**I5, I7, I12**	**I2, I4, I11**		3/6	??
Behaviour change skills		**I4**		0/1	0
SSB liking/preference			*C5, C6*	2/2	+
Child milk/water consumption	*C23*	P6	*C6*	1/3	0
Child TV viewing/screen time			*C5, C6, C11, C16, C17, C18, C21*	7/7	++
Child snack consumption			*C11*	1/1	+
Food fussiness		*C6*		0/1	0
(Taste) exposure		**I11**	**I5**	1/2	?
Interpersonal (parent/care giver)					
Family demographics					
Ethnicity (ref: white)		P6, *C2, C7, C8, C20, C16*	*C5, C22*	2/8	00
Parental age (ref: high)	*C13*	*C7*	*C2, C20, C25, C16*	4/6	++
Caregiver gender (ref: female)		*C2, C7*		0/2	0
Parents married/co‐habiting (ref: single)	*C2, C20*			2/2	−
Parent SES (ref: high)		P1, *C8, C13, C25*	*C2, C5, C7, C10, C19, C20, C16, C25*	8/12	++
Parental BMI/weight loss	**I13**	P4, *C12, C16*	*C20*	1/5	00
Maternal parity/N children		*C2, C8, C20*	*C25*	1/4	00
Parental psychosocial factors					
Parental knowledge	**I1, I5, I7, I9, I13,** *C4*	**I2, I3, I4, I6, I11**		6/11	??
Parental perceived barriers			**I1**	1/1	+
Parental attitude		**I10**		1/1	0
Parental perception of child's diet		*C21*		0/1	0
Parental self‐efficacy/motivation		**I6, I10,** C*4*		0/3	0
Parental support/encouragement	**I1, I9**	**I3, I11**, *C5, C24*		2/6	00
Parental behaviour					
Parental SSB consumption			P5, *C5, C14, C24*	4/4	++
Parental F&V consumption	*C24^F^*	*C24^V^*		1/2	?
Maternal (pregnancy) smoking		*C8, C25*	*C10, C20*	2/4	??
Maternal (pregnancy) sweet consumption		*C24*	*C20*	1/2	?
Parental food involvement/confidence		*C2, C21*		0/2	0
Parent/carer–child interaction					
Parenting skills	**I1, I5, I13,** *C4*	**I3, I6**, C24		4/7	??
Parental (positive) modelling	**I5, I13,** *C24*	**I9**, *C21*		3/5	− −
Parental monitoring		*C21*	*C5*	1/2	?
Formula fed (ref: breast fed)			*C10, C20, C25*	3/3	+
Early introduction to solids			P2, *C8*	2/2	+
Early introduction to SSB		P2		0/1	0
Parental rules/restriction/influence	*C11, C18*	*C21, C7*	*C24*	2/5	??
Pressure to eat		*C24*	*C21*	1/2	?
Using food as reward			*C18*	1/1	+
Verbal/material rewards	**I12**	*C24*		1/2	?
Environmental					
(Pre‐)school					
Attending out‐of‐home care			*C6, C9*	2/2	+
School policy	**I7, I8, I12**	**I2,** *C19*		3/5	− −
Staff knowledge	**I7, I8**	**I2, I11**		2/4	?
School water availability		**I2**		0/1	0
Staff skills	**I8**			1/1	−
School food availability		**I11**	**I12**	1/2	?
School cooking equipment		**I11**		0/1	0
Staff support	**I7**	**I11**		1/2	?
Home					
Home SSB/food availability		**I12**, *C3, C19, C21*	**I13**, *C5, C18*	3/7	??
Food security		*C3, C15*		0/2	0
Supermarket nearby	*C6*			1/1	−
Fast food/convenience store nearby			*C6*	1/1	+
Home location (ref: urban)		*C7*		0/1	0
Cost of F&V		*C21*		0/1	0
Community					
Raising general awareness/knowledge	**I7**	**I2**		1/2	?
Healthcare policy environment		**I6**		0/1	0

*Significantly lower/decreased SSB consumption; 0: no significant difference; +: significantly higher/increased SSB consumption.

**For **≤3 studies**: ‘0’: 0–33% of findings support association; ‘?’ 34–59% support association; and ‘+’ or ‘−’ if 60–100% support positive or negative association.

**For **≥4 studies**: ‘00’: 0–33% of findings support association; ‘??’ 34–59% support association; and ‘++’ or ‘− −’ if 60–100% support positive or negative association.

C, cross‐sectional studies – cross‐reference in Supplementary Tables 2–4; BMI, body mass index; F, fruit; V, vegetable; I, intervention studies; P, prospective studies; SES, socioeconomic status; SSB, sugar‐sweetened beverage.

#### Child‐level correlates/determinants

Ten individual (child)‐level correlates/determinants were investigated. There was evidence for a positive association with SSB consumption for SSB preference (2/2 studies: both XS), TV viewing/screen time (7/7 studies: all XS) and snack consumption (1/1 studies: XS). There was indeterminate evidence for child's age (5/10 studies reported a positive association: four prospective, six XS) and child's knowledge (protective effect in 3/6 intervention studies).

#### Interpersonal‐level correlates/determinants

Twenty‐eight interpersonal (parent/caregiver)‐level correlates/determinants were investigated. There was consistent evidence that lower parental socioeconomic (education, occupation or income) status (8/12 studies: one prospective, 11 XS), lower parental age (4/6 studies: all XS) and parental SSB consumption (4/4 studies: one prospective, three XS) were associated with higher SSB consumption in children. Parental (positive) modelling (3/5 studies: three intervention, two XS) was consistently associated with lower SSB consumption. There was some evidence that parents co‐habiting or being married (2/2: XS studies) was negatively associated, whereas parental perceived barriers (one intervention study), formula feeding (3/3: XS studies), early introduction of solids (2/2 studies: one prospective and one XS) and using food as a reward (1/1: XS study) was positively associated with SSB consumption. Parents' ethnicity, body mass index (BMI) and support or encouragement was consistently found to show no association with their child's SSB consumption.

#### Environmental correlates/determinants

Sixteen environmental correlates/determinants were identified. Within the preschool environment, most evidence came from four intervention studies. Together, they showed a positive influence of school policy on reduced SSB consumption (3/5 studies: four intervention, one XS). There was some evidence that attending out‐of‐home care was associated with higher SSB consumption (2/2 XS studies). The most studied correlate/determinant within the home environment was the home availability of SSB/food (in seven studies: two intervention, five XS), which showed an indeterminate/possible positive association (3/7 studies). There was some evidence from the same XS study that living near a supermarket was associated with lower consumption of SSB, whereas living near a fast food store was associated with higher SSB consumption. The influence of the healthcare policy environment was assessed in one intervention study, showing no effect on SSB consumption.

## Discussion

The results of this comprehensive review show that SSB consumption in young children is influenced by factors operating at individual, interpersonal and environmental levels, consistent with the socio‐ecological theory. Most determinants of SBB consumption that were targeted in the intervention studies included in this review were not studied in the observational studies identified, and this highlights the importance of synthesizing evidence from these different types of study designs together. Overall, intervention effect was indeterminate, with six of 12 multi‐level and parental interventions showing an effect on SSB consumption in the immediate post‐intervention period.

Parental modelling and SSB consumption were consistently associated with lower SSB consumption in children, suggesting positive parental modelling should be an important component of any intervention to reduce SSB consumption in young children (and their parents). However, none of the intervention studies identified included these as targets in their interventions. Similarly, parental feeding practices (formula feeding, early introduction of solids, using food as a reward, pressure to eat, perceived barriers) also appear to be important factors to consider in designing future interventions. Lower parental socioeconomic status, age and single parenthood were associated with higher SSB consumption. These findings are consistent with other research on the topic 82, 83.

We found that child‐level correlates such as TV viewing, snack consumption and preference for SSBs were positively associated with SSB consumption. This is in agreement with the clustering of this behaviours 82, 84, although the extent to which these associations are independent of each other or due to confounding is unclear. One factor might be the potential effect of TV advertising on promoting SSB consumption among young children. Surprisingly, there was no evidence that milk/water consumption was associated with lower SSB consumption and one intervention that targeted school water availability was not effective. The correlates/determinants studied most frequently at the child level were age (10 observational studies) and knowledge (six intervention studies). Both showed indeterminate/possible evidence and may warrant further investigation.

There was a consistent association with school nutritional policies and lower SSB consumption in young children aged 0–6 years. Two XS studies showed an association between SSB consumption and attendance at day care, although this could be due to higher SSB consumption at home. Among the environmental correlates, availability of SSB at home was shown by some studies to be positively associated with SSB consumption as was concluded by another recent study in obese/overweight Latino youth 85. The role of macro‐level environmental factors such as taxation, advertising/marketing, product price and placement have yet to be studied in relation to young children's SSB consumption. However, a recent systematic review suggested that taxation may not be effective in reducing SSB consumption in adults, and the impact on children may therefore be questionable 86.

A number of correlates/determinants, including child's and caregiver's gender, number of siblings, parental support, ethnicity and BMI, were consistently not associated with SSB consumption in young children. This suggests that the effectiveness of targeting interventions based on these factors is therefore likely to be minimal.

### Strengths and limitations of the review

Recent systematic reviews have highlighted the scarcity of evidence on the determinants of energy balance‐related behaviours in young children 27, 82, 83. To our knowledge, this is the first review of quantitative (interventional or observational) evidence on the determinants and correlates of SSB consumption in young children (0–6 years old).

Strict systematic review procedures were adhered to throughout the process to minimize reviewer‐related bias. No time or language restrictions were applied to ensure high sensitivity in identifying literature. Articles were hand searched and authors of included studies were contacted to identify grey and recent literature, resulting in four additional studies being included. We believe that we have included all relevant published studies (ranged from 1999 to 2014), although we are unable to rule out the possibility of publication and reporting bias 87. Also, the evidence identified came largely from economically developed countries. Our conclusions may therefore not be transferable to all countries, although increasing SSB consumption is also a public health concern in middle‐ and low‐income countries 88, 89, 90.

## Conclusions

Quantitative evidence supports several potential correlates/determinants of SSB consumption in young children operating at various levels of the socio‐ecological model. Most consistent evidence for potentially modifiable correlates/determinants was for parental modelling, child's TV viewing and school policy. Interventional evidence suggests that targeting parental‐, child‐ and environmental‐level determinants together could reduce SSB consumption in the immediate post‐intervention period.

## Conflict of interest statement

No conflict of interest was declared.

## Author contributions

VMP (lead reviewer) and RL (overall project lead and paired reviewer) contributed to all aspects of the review. HM (initial electronic database search in August 2012) and VMP (re‐run search in June 2014) were involved in identifying evidence using predefined search strategy. CO and KH screened (and CS, EMFvS and RL double screened) a proportion of abstracts and titles. EMFvS and KKO double reviewed all intervention studies and synthesized the evidence regarding potential determinants. All authors contributed to the study design, critical revision of the manuscript and approved the final version.

## Supporting information


**Table S1.** Extracted data of intervention and observational studies on SSB behaviour in young children.
**Table S2.** Description of intervention studies on sugar‐sweetened beverage consumption behaviours in young children, in chronological order.
**Table S3.** Description of prospective cohort studies on determinants of sugar‐sweetened beverage consumption in young children, in chronological order.
**Table S4.** Description of cross‐sectional studies on correlates of sugar‐sweetened beverages consumption in young children, in chronological order.
