# Supplementary material for: Determinants of sugar‐sweetened beverage consumption in young children: a systematic review
Source: Obes Rev. 2015 Aug 7;16(11):903–13. doi: 10.1111/obr.12310 (PMC4737242; doi:10.1111/obr.12310)
Supplement: Supplementary file 1 — Table S1. Extracted data of intervention and observational studies on SSB behaviour in young children. Table S2. Description of intervention studies on sugar‐sweetened beverage consumption behaviours in young children, in chronological order. Table S3. Description of prospective cohort studies on determinants of sugar‐sweetened beverage consumption in young children, in chronological order. Table S4. Description of cross‐sectional studies on correlates of sugar‐sweetened beverages consumption in young children, in chronological order. [file OBR-16-903-s001.docx]

**Table S1: Extracted data of intervention and observational studies on SSB behaviour in young children.**

| **Extracted data of intervention studies included:** | **Extracted data of non-intervention observational studies (CS and prospective cohort) included:** |
| --- | --- |
| Reference ID | Reference ID |
| Author | Author |
| Publication year | Publication year |
| Country | Country |
| Language of study | Study name |
| Study name | Sample size |
| Population | Age range |
| Sample size | Average age |
| Age range | Sex (M:F) |
| Average age | Ethnicity |
| Sex (M:F) | Socioeconomic status (SES) |
| Ethnicity | Study design |
| Socioeconomic status (SES) | Sample recruitment |
| Study design | Representativeness of sample |
| Sample recruitment | Follow-up period (for prospective cohort studies) |
| Representativeness (of sample to the general population i.e. not in specifically selected population such as children from low income background) | Level of determinant/correlate (socio- demographic, child behavioural, modifiable parental, environmental) |
| Intervention details | Primary outcome, (measured by) |
| Control group details | Secondary outcome, (measured by) |
| Setting (where the intervention was delivered) | Analysis type |
| Determinant targeted (child behavioural, modifiable parental, environmental) | Effect estimate (crude/ adjusted) |
| Theoretical model |  |
| Intervention provider |  |
| Number of sites |  |
| Duration of intervention |  |
| Follow-up |  |
| Primary outcome, (measured by) |  |
| Secondary outcome, (measured by) |  |
| Analysis type |  |
| Effect estimate (crude/ adjusted) |  |

**Table S2: Description of intervention studies on sugar sweetened beverage consumption behaviours in young children, in chronological order**

| **No** | **Author, year and country** | **Study design/**  **name** | **Population** | **Age at start** | **Setting** | **Intervention and provider** | **Targeted determinants**  **[theoretical model]** | **Duration of intervention**  **(follow-up if different)** | **Outcome** | **Measure** | **Effect** | **Quality score (max 8) and comments** |
| --- | --- | --- | --- | --- | --- | --- | --- | --- | --- | --- | --- | --- |
| I1 | Campbell 2013  Australia | Cluster-RCT  “InFANT” | NON-REP:54% university educated.  First time parent groups (int: 31, cont: 31).  N=542 at baseline  (Int: 271,Cont: 271)  N= 448 at 9month  N=389 at 20month | Mean 3.9 ± 1.6 months | Clinics & Parent groups | Parent-focused intervention on infants’ obesity-risk behaviours and BMI. Six 2-hr sessions incorporating a range of delivery modes and educational strategies including group discussion and peer support.  Delivered by trained dieticians. | **Parents** (knowledge, infant feeding & activity skills, barriers, social support).  [Parenting Support, Anticipatory guidance & Social cognitive theories] | 15 months  (outcomes at 9 & 20 month old) | SSB consumption  (% consumers) | 24-hour diet recall | ++ | Intermediate (5)  No allocation concealment, blinding and pre-intervention data of all outcomes |
| I2 | De Coen 2012  Belgium | Cluster-RCT  “Prevention of Overweight among Pre-school and school children (POP)” | REP: 31 schools across high, medium and low SES.  N=1589 at baseline  (int: 1032; cont:557)  N=694 at 2 year  (Int: 396 Cont: 298) | 2.5 to 6.5years | Pre-primary and primary schools | Health promotion programme with child at centre within several layers (family, friends, schools, community, stakeholders, local policy and media)  POP research team | **Multi-level**, incl.  **Child** (knowledge)  **Parents** (knowledge)  **School environment** (knowledge, policies, water availability)  **Community** (awareness)  [Socio-ecological theory] | 2 years | Soft drink consumption | FFQ | / | Intermediate (5)  Quasi- experimental design |
| I3 | Jones 2011  Australia | Uncontrolled pilot  “Time  2bHealthy” | NON-REP: Overweight preschool children and parents.  N=43 at baseline  N=40 at f/u | 2 to 5years | Home based | Interactive online parental education and discussion forums (5 modules, each module lasting 2 weeks) to promote healthy lifestyles in overweight preschool-aged children. | **Parents** (knowledge, parenting skills, social support).  [No theory identified] | 10 weeks | SSB consumption 1+ per month  (N, %) | Question-naire | † | Low  (Score N/A)  Uncontrolled pilot study |
| I4 | Llargues 2011  Spain | Cluster-RCT  “Avall” | REP: Children in the 1^st^ year of primary school, in Granollers, Spain.  N=372 at baseline (Int: 216, Cont:156)  N=230 at 2year  (Int: 190, Cont: 140) | 5 to 6years  (mean 6.3 ± 0.3years) | Schools | Promotion of healthy eating habits and physical activity through the educational method: Investigation, Vision, Action and Change (IVAC).  Delivered by school teachers trained in IVAC by specialist community project educators. | **Multilevel**, incl  **Child** (knowledge, skills to change behaviour),  **Parents** (knowledge).  [“Based on the principle that children are actors, able to operate over their environment”] | 2 years | 3+ fizzy drinks per week  (N, %) | FFQ, Krece Plus quick test ( to evaluate food habits of child) | / | Intermediate (5)  Allocation concealment, blinding data unclear and retention <70% |
| I5 | Stark 2011  USA | Pilot RCT “LAUNCH” | NON-REP: Children with BMI ≥ 95^th^% and 1+ overweight parent  N=18 (Int:8 Cont:10) | 2 to 5years  (mean 4.7 ± 1.1years) | Home & clinics | Designed to decrease or stabilize weight gain in obese preschool children. Specifically targeted preschool behaviours (e.g. food neophobia & tantrums for food).  Delivered by paediatricians & psychologists at parent-groups, child-groups & individual home visits. | **Multi-level**, incl.  **Child** (knowledge, taste, exposure)  **Parents** (knowledge, parenting skills, parental modelling)    [Social Cognitive Theory] | 6 months  (outcomes at 6 & 12 months) | SSB/high calorie drink consumption | 24 hour recall | †† | Low  (Score N/A)  Very small pilot study |
| I6 | Taveras 2011  USA | Cluster-RCT  “High Five for Kids” | NON-REP: Children with BMI ≥ 95^th^% and 1+ overweight parent.  N=275 at baseline (Int: 271, Cont: 204)  N=445 at 1 year  (Int: 253, cont: 192) | 2 to 6.9years  (mean 5.2 ± 1.1years) | Primary care paediatric clinics | Obesity intervention. Families received motivational interviewing (4x25-min visits & 3x15-min phone calls) and educational modules targeting TV viewing and fast food and SSB consumption.  Primary care paediatric nurse practitioners were the key intervention deliverers. | **Multilevel**, incl.  **Parents** (knowledge, self-efficacy, motivation, skills), **Health Care Environment** (policy environment)  [Chronic Care Model] | 1 year | SSB consumption | FFQ | † | High (6) |
| I7 | De Silva-Sanigorski 2010  Australia | Quasi-experimental  “Romp and Chomp” | REP: Targeted ~12,000 children in 2004 – 2008  N=1000-1265 per sample were assessed | 2 or 3.5years | Early-childhood  care and educational settings | Community wide obesity prevention in 0-5 year-olds by promoting healthy eating and active play in children  < 5y.  Planned and implemented by several key organisations (incl. medical and dental health service providers, local and state governments, pre-schools, & the regional sporting co-ordinating body). | **Multi-level**, incl.  **Child** (knowledge)  **Parents** (knowledge)  **Pre-schools, Centre- & Home-based day-care environments** (knowledge, policies)  **Community** (awareness, support for preschool staff)  [No theory identified] | Up to 4 years (outcome based on Year 3 data collected in 2008) | Cordial (sugar syrup) consumption | Question-naire (EPAQ) | †† | Intermediate (4)  Repeated sampling pre and post intervention and in comparison communities. |
| I8 | Hardy 2010  Australia | Cluster-RCT  “Munch and Move” | REP: Preschool children  N=334 at baseline (int: 207, cont: 127)  N=213 at 20 weeks  (Int: 213, cont:134) | 4.4 to 4.9y  (mean 4.5 ± 0.3years) | Preschools and day care centres | A low-intensity, sustainable professional development program to promote strategies within preschool and child care centres that encourage children’s healthy eating, active play, and fundamental movement skills. Delivered by preschool & child care centre staff. | **School/Centre environment** (preschool healthy eating policy, staff knowledge, staff skills)  [No theory identified | 20 weeks | SSB servings | Objective evaluation  of children’s lunchbox contents | †† | High (6) Study measured drinks serves |
| I9 | Watt 2008  &  Scheiwe 2010  UK | RCT  “Infant feeding peer support trial” | NON-REP: New mothers from disadvantaged neighbourhoods  N=312 at baseline (Int 157, cont: 155)  N=239 at 12 months  (Int: 115, Cont: 124)  N=101 at 4-5 years  (Int: 55, Cont: 46) | ≤ 12 weeks at start | Home based | Monthly home visits to empower new mothers to follow current guidance on infant feeding, in particular, to increase vitamin C consumptions from fruit.  Delivered by trained volunteers (local mothers). | **Parents (mothers)** (knowledge, self-efficacy, social support).  [Social support theoretical model] | 9 month  (outcomes at 12 & 18month old, and 4-5year old) | Squash consumption | FFQ, Question-naire, & interview | †  12 & 18mo  ++  4-5y | High (6) |
| I10 | Whaley 2010  USA | Non-randomised trial  "Child health and intervention research project” (CHIRP) | NON-REP: Low-income mothers (94% Latino)  N=821 at baseline  (Int: 412, Cont: 409)  N=589 at 12months  (Int: 293, Cont: 297) | 1 to 5years  (mean 23 ± 9.2months) | 1 WIC site in Pomona CA, USA | Enhanced education on healthy nutrition to parents at their 6 monthly WIC recertification appointments.  Delivered by WIC staff using motivational interviewing techniques. | **Parents** (self-efficacy, attitude)  [Trans Theoretical Model] | 12 month | SSB consumption (times/day) | Question-naire | + | High (6)  Scored 6 due to non –randomisation and subjective assessment of outcomes. |
| I11 | Vereecken 2009  Belgium | Cluster-RCT  “Beastly healthy at school” | REP: 16 schools (int: 8, cont: 8)  N=1063 at baseline  (int: 618, cont: 445)  N=476 at 6 months  (int: 308, cont: 168) | NA | Preschools | Intervention aimed to assist preschools to implement a healthy school food policy. The main objectives were to increase consumption of fruit, vegetables and water and to decrease the consumption of sugared milk drinks and fruit juice.  2 day training to school staff.  Educational material for staff, parents and children. | **Multi-level**, incl.  **Child** (knowledge, food exposure)  **Parents** (knowledge, support)  **School environment** (carer knowledge, food availability, cooking equipment availability, support)  [No theory identified] | 6 months | SSB & Sugared milk consumptions per day | FFQ, parent report, teacher audit | † | Intermediate (4)  No allocation concealment, blinding and pre-intervention data of all outcomes |
| I12 | Korwanich 2008  Thailand | Cluster-RCT | REP: Nursery schools in rural North Thailand (int: 8, cont:8)  N=219 at baseline and at 9 months  (Int: 135, cont: 84) | 2 to 5years | Nursery schools | New healthy eating school food policies were developed in each school by parent representatives, educational experts, teachers, school board members and health workers. Most policies aimed to decrease consumption of crispy and sugar-containing snacks, and to provide healthy snacks. Roles and responsibilities were given to all stakeholders: teachers, school board members and parents. | **Multi-level**, incl.  **Child** (knowledge, reward)  **Parents** (food provision/ catering)  **School environment** (preschool healthy eating policy &food availability in canteens & shops)  [No theory identified] | 9 months | SSB consumption in school (frequency) | Observed school consump-tions, collected food wrappings | †† | High (6)  Authors described study as ‘quasi-experimental’ as other determinants were not controlled. |
| I13 | Klohe-Lehman 2007  USA | Uncontrolled trial | NON-REP: Low-income, overweight or obese mothers (62.6% Hispanic).  106/235 completed the intervention  N=91 with outcome | 1 to 3years  (mean 2.1years) | Public health clinics / groups | Weight loss intervention for mothers (8x weekly 2-hr classes: 15-min weigh-in, 1.25-hr discussion and activities, 30-min exercise).  Delivered by registered dieticians. | **Multilevel**, incl.  **Parents (mothers)** (knowledge, modelling, parenting skills, maternal weight loss)  **Home environment** (home availability of SSB)  [Social Cognitive Theory] | 8 weeks | SSB consumption | FFQ | †† | Low  (Score N/A)  Uncontrolled pilot study |

SSB: Sugar sweetened beverage; RCT: Randomised control trial; REP: Representative; NON REP: Non representative; BMI: Body Mass Index; Int: Intervention group; Cont: Control group; f/u: Follow up; FFQ: Food frequency questionnaire; N/A: Not applicable; WIC: Women, Infants and Children; ++: statistically significant positive effect of intervention; + or - : statistically non- significant positive or negative effect of intervention; /: no effect of intervention.**Table S3: Description of prospective cohort studies on determinants of sugar sweetened beverage consumption in young children, in chronological order**

| **No** | **Author, year and country** | **Study design/**  ***name*** | **Population** | **Age range of study population** | **Setting** | **Follow-up period** | **Determinant** | **Outcome** | **Measure** | **Association** | **Quality score (max 6) and comments** |
| --- | --- | --- | --- | --- | --- | --- | --- | --- | --- | --- | --- |
| P1 | De Coen 2012  Belgium | Prospective cohort of cluster-RCT  *“Prevention of Overweight among Pre-school and school children (POP)”* | REP: 31 schools across high, medium and low SES.  Only observational data of control group reported here.  Total number of children at baseline, cont:557 and at 2 year cont: 298 | 2.5 to 6.5 years | Pre-primary and primary schools | 2 years | **Socio-demographic**: SES | Soft drinks consumption (ml/day) | FFQ | / | Intermediate (4)  Intervention study with quasi- experimental design based on Nutrition and PA targets of Flemish Community using socio-ecological model. |
| P2 | Koh 2010  Australia | Prospective cohort  *Perth Infant Feeding Study II (PIFS II)* | REP - 587 Post -partum women and children recruited at hospitals soon after delivery. | Birth to 52 weeks | Home based | 52 weeks | **Parental:** Early introduction to solids  **Parental:** early introduction to SSB (between 22 weeks and 52 weeks) | Introduction to fruit juice by 22 weeks  Pattern of increased consumption of fruit juice , cordial and soft drinks in infants | Question-naire and telephone interview | ††  + | Intermediate (4)  Subjective outcome measures.  Fruit juice is non- core food in this study, as it increased contribution to total sugar and energy consumption in children’s diet  The association between age and exposure to fruit juice, cordial and soft drinks appears to be significant. |
| P3 | Lim 2009  USA | Prospective cohort  *Detroit Dental Health Project* | NON REP- 365 low income, African American, preschool children (boys: 48.4%). | 3 to 5 years | dental clinic based | 2 years | **Socio-demographic**: Childs age | soda consumption    fruit drink consumption (excludes 100% fruit juice)  all SSB consumption | Block kids food frequency question-naire | -  †  † | Low (2)  Non representative sample and subjective outcome measures |
| P4 | Kral 2008  USA | Prospective cohort | NON REP - 49 healthy, full term infants, ethnicity - white. | 3 months to 6 years | home based | Each year from age 3 to 6 | **Socio-demographic**: Childs age(consumption pattern from ages 3 to 6)  **Parental:** high maternal pre-pregnancy BMI | Fruit juice consumption  Soda consumption  Soft drinks including fruit juice consumption  Beverage (soda, diet soda, soft drinks and fruit juice) consumption pattern  Beverage (soda, diet soda, soft drinks and fruit juice) consumption pattern | 3 day food records | †† in high risk group at ages 3 and 4  †† in high risk group at age 6  †† in high risk group at ages 3, 4 and 5  †† for soda at age 6  †† for fruit juice at ages 3 and 4  †† for soft drinks at ages 3, 4, and 5  Generally † association in high risk group. | Low (2) small study in non- representative sample. |
| P5 | Hoerr 2006  USA | Cohort data of intervention study  *Early Head Start* | NON REP – 93 mother toddler dyads from families with limited incomes. 78.6% of sample was Caucasians. | 12 months to 36 months | Home based | When child was 24 months and when child was 36 months old | **Socio-demographic**: Childs age  **Parental:** Mother's soft drink consumption/negative modelling | Soft drink consumption  Sweetened beverage consumption  caffeinated beverage consumption  Child's soft drink consumption | 24 hour dietary recall | +  -  /  †† | Low (2)  Results of analysis of combined data from both “Early Head Start” Int: 51 and cont: 42 |
| P6 | Ute Alexy 1999  Germany | Prospective cohort  *Dortmund Nutritional and Anthropometric Longitudinally Designed Study (DONALD)* | NON REP: 205 preschool children (boys: 51%) from health conscious families volunteered to participate in the study. | 3 to 5 years | Study centre | 2 years | **Socio-demographic**: Childs age (from 3 to 5 years)  **Child**: fruit juice consumption | Sweetened beverage consumption  Added sugars (sum of added monosaccharaides, disaccharides and oligosaccharides) consumption through low and excessive fruit juice consumption  Sweetened beverage consumption | 3 day weighed diet record | +  +  - | Intermediate (4)  Non representative population - health conscious participants in study |

SSB: Sugar sweetened beverage; RCT: randomised control trial; REP: Representative; NON REP: Non representative; BMI: Body Mass Index; Int.: Intervention group; Cont.: Control group; FFQ: Food frequency questionnaire; PA: Physical activity; ml: millilitres; SES: Socio economic status; ++ or - - : statistically significant positive or negative association; + or - : statistically non- significant positive or negative association; /: no association.

**Table S4: Description of cross sectional studies on correlates of sugar sweetened beverages consumption in young children, in chronological order**

| **No** | **Author, year and country** | **Study design/ name** | **Population** | **Age range** | **Recruitment Site** | **Correlate** | **Behaviour** | **Measure** | **Association** | **Quality score (max 6) and comments** |
| --- | --- | --- | --- | --- | --- | --- | --- | --- | --- | --- |
| C1 | Kong 2013  USA | XS | NON REP – 331 children and 352 mothers of African American or Hispanic ethnicity, from 12 WIC sites in Chicago. | 2 to 3.5 years | Community based | **Socio-demographic:** Parent ethnicity (African American V/s Hispanic) | SSB consumption | 1 valid 24 hour recall | ++ | Intermediate (3)  XS study design, non- representative sample, analysis  . |
| C2 | Ohly 2013  UK | XS | NON REP – 394 children and their parents from low income families | 18 to 60 months | Home based | **Socio-demographic:** Parent gender male  **Socio-demographic:** Parent ethnicity white British  **Socio-demographic:** Parent marital status married/cohabiting  **Socio-demographic:** Parent unemployed  **Socio-demographic:** Parent Education (none/GCSE)  **Socio-demographic:** Parent receiving benefits  **Socio-demographic:** Older parent s  **Socio-demographic:** Number of children  **Socio-demographic:** Childs age  **Parental:** Parental food involvement | Sugary drinks consumption | Multiple pass 24 hour recall | +  /  ‒ ‒  +  ++  +  ‒ ‒  +  ++  - | Low (2)  XS study design, non- representative sample, and subjective outcome measures. |
| C3 | Bauer 2012  USA | XS of cRCT  *Bright start study* | NON REP- 432 dyads of parents or caregivers of kindergarten age children living on a rural American Indian reservation | Kinder-garten age | School based | **Environmental:** Food security  **Environmental:** Home food availability | SSB consumption | Parent report and 6 item short form of household food security scale | /  / | Low (1)  XS study design, non- representative sample, subjective outcome measures and analysis. |
| C4 | Goodell 2012  USA | XS | NON REP – Convenience sample of 198 parents from low socioeconomic status recruited at local health fairs in Connecticut, served by WIC offices. | 1 to 5 year old | WIC Clinics | **Parental:**  Motivation  **Parental:**  Knowledge  **Parental:**  Behavioural skills | SSB consumption | Parents completed home beverage inventory and information –motivation-behavioural (IMB) skills survey | /  ‒ ‒  ‒ ‒ | Low (1)  XS study design, non- representative sample, subjective outcome measures and analysis. |
| C5 | Mc Gowan 2012  UK | XS  *Poppets Study* | REP – 434 primary caregivers of children from preschools and children’s centres in London | 2 to 5 years | Community  based | **Socio-demographic:** child’s age  **Socio-demographic: C**hild’s gender  **Socio-demographic:** Parental education high  **Socio-demographic:**  Ethnicity (white)  **Parental:** Maternal consumption of SSB  **Parental:** Encouragement  **Parental:** Monitoring  **Child :** Child liking item  **Child:** TV viewing  **Environmental:** Home availability of SSB | Non -core drink  (sweetened carbonated drinks, diet sweetened carbonated drinks, other sweetened drinks) | CFQ (child feeding questionnaire) and PFSQ (parental feeding style questionnaire) | ++  +  - -  ++  ++  -  ++  ++  ++  ++ | Low (2)  XS study design, exposure and subjective outcome measures  Unclear if boys or girls consumed more SSB. However, there were 54% girls in the analysis |
| C6 | Pabayo 2012  Canada | Prospective cohort  with cross sectional analysis  *Spatial Health Assessment of Pre-schooler’s*  *Environments (SHAPEs)* | REP -Data collected from 1179 children attending a public health unit for immunization in Edmonton region, Canada | 4 - 5 years | Public health unit for immunization | **Socio-demographic:** child’s gender: Boys  **Socio-demographic:** SES low or medium  **Socio-demographic:** child’s age: 5 v/s 4  **Child:** High screen time  **Child:** High desire to drink  **Child:** Milk consumption  **Child:** Water consumption  **Child:** Fussy with food  **Environmental:** Attending day care  **Environmental:** Grocery store near residence  **Environmental:** Fast food or convenience store within 1 km of residence | Soft drink / fruit juice consumption | General demographics survey questionnaire, Children’s Eating Behaviour Questionnaire (CEBQ), an instrument developed by nutritionists in the public  health authority was used to assess dietary consumption of the  children | ++  ++  ++  ++  ++  ++  ++  ++  ++  ++  ++ | Intermediate (4)  XS study design, subjective outcome measures. |
| C7 | Erinosho 2011  USA | XS  *2007 California Health Interview Survey (CHIS)* | REP- 1105 children and their care givers from a publicly available dataset of CHIS | 3 to 5 years | Community based | **Socio-demographic:** Caregivers gender male  **Socio-demographic:** Caregivers ethnicity Hispanic  **Socio-demographic:** Caregivers age >35 years  **Socio-demographic:** caregivers education college  **Socio-demographic:** caregivers residence rural  **Parental:** caregiver has some influence over child’s eating | SSB consumption | Short dietary questionnaire with 8 questions and caregiver report on previous day food consumption | +  +  -  - -  -  + | Intermediate (3)  XS study design, exposure and subjective outcome measures |
| C8 | Koh 2010  Australia | Prospective cohort study  with cross sectional analysis  *Perth Infant Feeding Study II (PIFS II).* | REP –453 Post -partum women and children recruited at hospitals soon after delivery. | Birth to 52 weeks | Home based | **Socio-demographic:**  Maternal country of birth Australia, UK/ Ireland (vs other)  **Socio-demographic** : Maternal education  **Socio-demographic:** Maternal parity  **Parental:** Maternal smoking during pregnancy  **Parental:** introduction of solids in infants at < 17 weeks | Early introduction of Fruit juice, cordial and soft drinks (by 52 weeks) | Questionnaire and telephone interview | + for all SSB  / for fruit juice  † for cordial & soft drinks  + for fruit juice  + for cordial & soft drinks  † † for cordial & soft drinks | Intermediate (3)  XS study design and subjective outcome measures.  Fruit juice is non- core food in this study, as it increased contribution to total sugar and energy consumption in children’s diet |
| C9 | Lehtisalo 2010  Finland | XS  Nutrition study within the *Type 1 Diabetes prediction and prevention birth cohort study (D1PP)* | REP- 471 Finnish children in day care and cared at home | 3 year olds | Community based | **Environmental:** Cared at home v/s  Day care outside home | Beverages consumption (juice and soft drinks) | 3 day food record | + for juice drinks and  ++ for soft drinks | Intermediate (3)  XS study design, subjective outcome measures and analysis. |
| C10 | Schiess 2010  5 European countries (Belgium, Germany, Italy, Poland and Spain) | XS of RCT  *European childhood obesity project* | REP – 1368 healthy, singleton, term infants | Birth to 12 months | Home based | **Socio-demographic:** Low maternal education  **Socio-demographic:** Child’s country of birth  Italy, Poland, Spain, Belgium  **Parental:** Maternal smoking  **Parental:** Formula feeding  v/s Breast feeding | SSB - introduction of instant tea and energy providing liquids (EPL) in infants by 3 months  SSB- introduction of fruit juice or EPL at 1-6 months  SSB- introduction of EPL during first 4 months of life in infants  SSB-early introduction of EPL (during first 4 months in infants) | Monthly ( 1-9 months) and at 12 months, weighed 3-day food protocol on 3 consecutive days i.e. 2 week days and 1 weekend day. | ++  ††  ††  †† | Intermediate (4)  XS study design, subjective exposure and outcome measures  EPL includes sugared instant tea, fruit juice, fruit drinks, veg juice, other sweetened beverages (soft drinks and flavoured or unflavoured sugared water)  Reference country: Germany |
| C11 | Gubbels 2008  Netherlands  Gubbels 2009 | XS of cohort study  *KOALA birth cohort study*  *KOALA is an acronym (in Dutch) for:*  *Child, parents and health: lifestyle and genetic constitution.* | REP- 1931 children in 2008 analysis  2276 children in 2009 analysis | 2 year olds | Community based | **Parental:** Prohibition of snack and soft drink consumption  **Parental:** Absence of rules re: cookies and cake consumption  **Child:** TV viewing  **Child:** Snack consumption | Sugared soft drinks  Consumption  Sugar sweetened drinks/ soft drinks consumption | 65 item FFQ | ‒ ‒  + +  ++  ++ | Intermediate (3)  XS study design, subjective outcome measures |
| C12 | Lim 2009  USA | XS analysis of longitudinal Cohort data.  *Detroit Dental Health Project.* | NON REP- 365 low income, African American, preschool children. | 3-5 years | Dental clinic based | **Socio-demographic:** Caregiver's BMI  **Socio-demographic:** Child’s gender male | Soda, fruit drinks and SSB consumption | Block kids food frequency questionnaire | † for soda and all SSB consumption  ††for fruit drink consumption  + | Low (2)  Non representative sample and subjective outcome measures. |
| C13 | Navia 2003  Spain  Navia 2009 | XS | REP- 110 preschool children from 2 day care centres in Madrid included in the 2003 analysis  103 preschool children included in the 2009 analysis | 2 to 6 years | day care centre based | **Socio-demographic:** Maternal education middle/high  **Socio-demographic:** Maternal age > 26 years | SSB- Non- alcoholic beverages | Food monitoring (“precise individual weighing” of served and left over food ) by trained personnel | +  + + | Intermediate (3)  XS study design, exposure and analysis  Children from younger mothers (<26 yrs.) consumed significantly less sugars and alcohol free beverages |
| C14 | Papas 2009  USA | XS of intervention study (longitudinal RCT) | NON REP- 109 low income, African American, adolescent, primiparous mothers | Birth to 2 years | Home based | **Parental:** Maternal soda consumption (at least 2 sodas a day) | SSB- soda consumption in child | Toddler diet measured by 73 item feeding check list  Mothers diet measured by youth Adolescent FFQ (YAFFQ) | ++ | Intermediate (3)  XS study design, non- representative sample.  Study reports that association is not moderated by intervention status |
| C15 | Rosas 2009  USA and Mexico | XS  *Centre for the health assessment of mothers and their children of Salinas (CHAMACOS) longitudinal birth cohort study* | NON REP- Parents and children of Mexican descent, living in migrant communities in California (n=301) and Mexico (n= 301) | 5 year olds | Community based | **Environmental:** Food security | SSB consumption | 2 types of FFQ’s – one to account for differing food environments | / | Intermediate (3)  XS study design, non- representative sample, and subjective outcome measures. |
| C16 | Dubois 2007  Canada  Dubois 2008 | XS of prospective cohort  *Longitudinal study of Child development in Quebec* | REP- 1549 preschool children  REP – 1549 children in Quebec. | 5 months to 5 years  4 to 5 years | Home based  Public health clinic | **Socio-demographic:** maternal education low  **Socio-demographic:** income low  **Socio-demographic:** maternal age  **Socio-demographic:** mother immigrant  **Socio-demographic:** child’s gender male  **Parental:** parental overweight/obese  **Child:**  TV viewing | SSB consumption from 2.5 to 4.5 years  SSB consumption | 24 hour dietary recall interview and FFQ at2.5, 3.5 and 4.5 years  24 hour diet recall, an eating behaviour and TV viewing questionnaire | + +  ++  ‒ ‒  †  †  /  ++ | Intermediate (3)  XS study design and subjective outcome measures.  SSB consumption high in households with less than $20,000annual income  SSB consumption high when mothers were immigrant, younger than 25 y, with low education |
| C17 | Miller 2008  USA | XS  *VIVA project* | REP- 1203 mother child dyads participating in VIVA project | 3 year olds | Clinic based | **Child:** TV viewing | SSB consumption | FFQ | + + | Intermediate (3)  XS study design in mothers, subjective outcome measures |
| C18 | Spurrier 2008  Australia | XS | REP- 280 preschool children from southern metropolitan Adelaide | Under 6 years  Mean 4.8 years | Home based | **Parental:** One or both parents eat main meal with children  **Parental:** Reward good behaviour with food  **Parental:** Restrict carbonated drink/cordial  **Parental:** Restrict fruit juice  **Child:** Evening meal in front of TV  **Environmental:** availability of fruit juice or cordial and carbonated drink in home | SSB consumption | Physical and nutritional home environment inventory, parent report and direct observation | -  + +  - -  ‒ ‒  + +  + + | Intermediate (3)  XS study design, exposure and outcome measures |
| C19 | Vereecken 2008  Belgium | XS | REP- 1500 Flemish preschool children (and their parents) from 50 nursery schools | 2.5 to 6.5 years | School based | **Socio-demographic:** Child’s gender girl  **Socio-demographic:** Childs age  **Socio-demographic:** Maternal education high  **Socio-demographic:** Paternal education high  **Environment:** Sugared milk availability  **Environment:**  School food policy | Sugared milk drink consumption | FFQ | +  -  - -  -  +  + | Intermediate (3)  XS study design, exposure and outcome measures |
| C20 | Brekke 2007  Sweden | XS of prospective, population-based, longitudinal cohort study.  *All Babies In Southeast Sweden (ABIS)* | REP – 10,762 children examined when they were between 9 and 18 months. Diet diaries were available for 9849 children | Birth to 18 months | Home based | **Socio-demographic:** Child with older siblings  -----------------------------  **Socio-demographic:** Mother <25 years old when giving birth  **Socio-demographic:** Father <25 years old when child was born  **Socio-demographic:** Mother living alone at time of birth of child  **Socio-demographic:** Paternal education low  **Socio-demographic:** Maternal education low  **Socio-demographic:** Father from Sweden  **Socio-demographic:** Mother from Sweden  **Parental:** Duration of exclusive breastfeeding short  **Parental:** High maternal consumption of sweets during pregnancy  **Parental:** Maternal smoking during pregnancy  **Parental:** Maternal BMI >25 at 1-year examination  **Parental:** Paternal BMI >25 at 1-year examination | Introduction of  sugar-sweetened drinks at day 240 or earlier | Follow-up questionnaires from 10 762 children at 1 year of age and detailed food diaries of 9849 parents and children | +  + +  +  + +  +  + +  -  -  ++  + +  + +  + +  + | Intermediate (4)  XS study design and subjective outcome measures. |
| C21 | Campbell 2006  Australia | XS | REP- 560 children and their primary care providers (mothers 92% and fathers 8%) from 3 socio economically distinct areas | 5 to 6 year olds.  Mean age 6.1 years | School based | **Parental:** Perception of adequacy of child’s diet  **Parental**: modelling of eating  **Parental:** Restriction of eating  **Parental:** Monitoring of eating  **Parental**: low preference for fruit and vegetables  **Parental:** Pressure to eat  **Parental:** Confidence in cooking  **Child:** TV viewing  **Child:** Mealtime interruptions  **Environmental:** Food availability  **Environmental:** High cost of fruit and vegetable | SSB- high energy, non-dairy drink consumption | 56 item FFQ | +  -  -  -  +  ++  -  ++  +  +  + | Intermediate (3)  XS study design, mainly mothers, subjective outcome measures |
| C22 | Mennella 2006  USA | XS  *Feeding Infants and Toddlers study (FITS)* | REP – 371 Hispanic and 2637 non-Hispanic infants and toddlers | 4-5 months, 6-11 months 12-24 months | Home based | **Socio-demographic:** Parent ethnicity (Hispanic V/s Non-Hispanic)  **Socio-demographic:** Childs age | SSB consumption | 24 hour diet recall | ++  ++ | Low (2)  XS study design, analysis and subjective outcome measures |
| C23 | Marshall 2005  USA | XS of longitudinal study  *Iowa Fluoride Study* | REP – 399 participants (mothers & new-born’s from Iowa fluoride study) | Birth to 5 years | Home based | **Environmental:** Age  from 1 to 5 years  **Child:** milk consumption | Added sugar beverage consumption  Juice drink, soda pop and sugar beverages consumption | 3-day food and beverage diaries at 1, 2, 3, 4 and 5 years | +  ‒ ‒ | Intermediate (3)  XS study design, subjective exposure and outcome measures |
| C24 | Vereecken 2004  Belgium | XS | REP- 316 children (and their parents) from 6 preschool kindergartens | 2.5 to 7 years  Mean 4.7 years | Pre-school  based | **Parental:** Mother frequent consumer  **Parental:** Permissiveness  **Parental:** Pressure  **Parental:** Material reward  **Parental:** Verbal praise  **Parental:** Negotiation  **Parental:** Encourage fruit consumption  **Parental:** Encourage veg consumption  **Parental:** Discourage soft drinks consumption  **Parental:** Discourage sweets consumption  **Parental:** Catering on demand  **Parental:** Restrain from negative modelling  **Parental:** Mothers fruit consumption  **Parental:** Mothers vegetable consumption  **Parental:** Mothers soft drink consumption  **Parental:** Mothers sweet consumption | Soft drink consumption | Short four item FFQ compared with 3 day food diary of 2 weekdays and 1 weekend day in sub sample n= 159 | ++  ++  -  +  ‒ ‒  -  -  +  +  +  ++  ‒ ‒  ‒ ‒  -  ++  + | Intermediate (4)  XS study design, subjective outcome measures |
| C25 | Johnson 2007  UK  Northstone 2002  Rogers 2003 | Prospective Cohort  with cross sectional analysis of ( CIF – children in focus) AVON longitudinal study of parents and children (ALSPAC)  XS / sub study *(CIF – children in focus) of*  *AVON longitudinal study of parents and children (ALSPAC)*  XS / sub study *(CIF – children in focus) of*  *AVON longitudinal study of parents and children (ALSPAC)* | NON REP – 521 preschool children mainly of white and affluent background  REP-1026 toddlers participating in the ALSPAC study  REP- 993 toddlers participating in the ALSPAC study | 5 years  18 month olds  18 month olds | Community  based  Clinic based  Community based | **Socio-demographic: Maternal education**  **Socio-demographic:** Maternal education low  **Socio-demographic:** Maternal age  **Socio-demographic:** Number of older siblings  **Socio-demographic:** major financial problems  **Socio-demographic:** housing tenure vs non-council  **Parental:** Maternal smoking  **Parental:**  Formula-feeding vs breastfeeding  **Socio-demographic:** Maternal age <25  **Socio-demographic:** Maternal education low  **Parental:** Maternal smoking | SSB consumption  Low calorie squash, orange juice, squash, Tea/coffee, black current, apple juice, flavoured milk, fizzy drinks, baby drinks, formula milk consumption  Tea, sugar sweetened soft drink and diet soft drink consumption | Diet diaries  24 hour recall  3 day food record, questionnaire | /  ++  - -  ++  ++  ++  /  ++  +  +  ++ | Low (2)  XS study design, non- representative sample, subjective outcome measures and analysis.  Intermediate (4)  XS study design and outcome measures. Not clear from study that it is non representative |

XS: Cross sectional; WIC: Women, infant, child; SSB: Sugar sweetened beverage; cRCT: Cluster randomised control trial; XS: Cross sectional study design; REP: Representative; NON REP: Non representative; BMI: Body Mass Index; FFQ: Food frequency questionnaire; CI: Confidence interval; OR: Odds ratio; IQR: Interquartile range; ++ or - - : statistically significant positive or negative association; + or - : statistically non- significant positive or negative association; /: no association.
